# Linking current dental education to gerontological education to meet the oral health needs of growing aging populations

**DOI:** 10.3389/froh.2023.1232489

**Published:** 2023-10-09

**Authors:** Maryam Tabrizi, Wei-Chen Lee

**Affiliations:** ^1^ School of Dental Medicine, University of Nevada, Las Vegas, NV, United States; ^2^ Department of Family Medicine, John Sealy School of Medicine, University of Texas Medical Branch, Galveston, TX, United States

**Keywords:** dental education, oral health, 4Ms, age-friendly, aging population

## Abstract

**Objective:**

This study aimed to recognize the gaps in dental education by studying the current level of geriatric oral health training of recent graduated dentists who have been admitted into an Advanced Education in General Dentistry (AEGD) program.

**Methods:**

The AEGD program was developed along with the Age-Friendly 4Ms model to enhance current dental education. We adopted the Rapid Cycle Quality Improvement model to test the effectiveness of the training for AEGD residents from 2019 to 2022. A total of 18 residents participated (6 residents each year). A 5-question survey was administered before and after the rotation and Wilcoxon signed-rank with Fisher Exact tests were conducted to compare pre- and post- rotation results.

**Results:**

All 18 residents have completed pre- and post-program surveys. They self-reported minimal to no training in preparation to provide care to older adults with multiple chronic conditions. After the rotation, residents’ confidence in treating older adults was significantly increased (*p* = 0.011). Meanwhile, residents gained knowledge to apply the 4Ms framework (what matters, medication, mentation, and mobility) to their practices (*p* = 0.015) and provide age-friendly care for older adults.

**Conclusion:**

The study identified and addressed the missing link in dental education to gerontological and geriatrics education. More clinical rotations and didactic training to equip residents with competences of providing geriatric oral health are strongly recommended.

## 1. Introduction

The aging population of the United State has changed the demographic distribution from a pyramidal shape to the shape of a pillar (1). Baby boomers are the individuals born after WWII between 1946 and 1964 and the first cohort of boomers born in 1946 have turned 64 in 2011. According to U.S Census in 2018, the population landscape has changed as about 10,000 boomers will turn 65 every day until 2030 (2). By 2030, the number of older adults over the age of 65 will increase to more than 74 million in which approximately 30% (22 million) will need special care for complex medical conditions (3). Humans are living longer, which is accompanied by a rising number of chronic conditions including oral conditions (4). Building workforce to meet the increasing needs for caregivers and health services became one of the national priorities (5). The demand for geriatric health and oral health is high and will go higher if we fail to take actions now. Therefore, to mitigate the burden of oral health among an aging population requires recognizing the missing link between training of dental professionals and real-world practices and strategically building a bridge between oral health care and overall health care (6).

**Figure 1:**
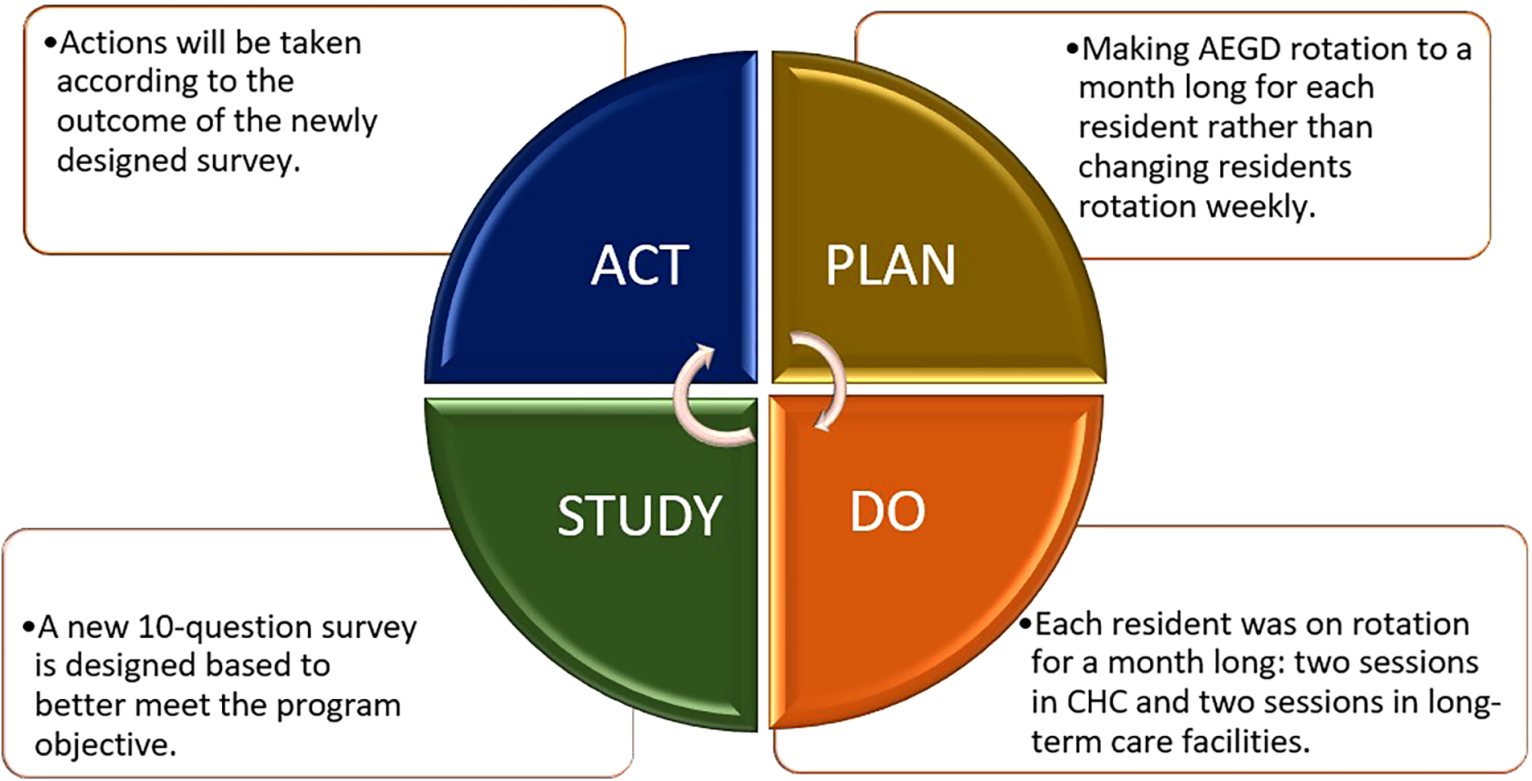
Steps of the rapid cycle to improve the advanced education in general dentistry.

The predoctoral Commission on Dental Accreditation (CODA) Standards 2–25 requires graduates to be competent in assessing and managing the treatment of patients with special needs such as the vulnerable elderly (7). However, teaching geriatric dentistry is not yet standardized and imbedded within dental education in majority of dental schools resulting in inadequate access to oral health services among older adults (8–10). Significant barriers to training dental students adequately to treat geriatric patients with multiple health frailties have been discussed (11–13). Barriers to adequate training include curriculum, funding, and lack of full-time faculty to teach didactic and clinical courses in geriatric dentistry. Meanwhile, post-graduate dental (PGD) training is not required for practice and most PGD programs heavily rely on federal investments (14, 15). There is no standard guideline to train faculty members to establish a postdoctoral or residency curricula either. In response, we developed an Advanced Education in General Dentistry (AEGD) program while using the Rapid Cycle Quality Improvement (RCQI) with four sections Plan-Do-Study-Act to make necessary changes before the next cycle (Figure 1) (16).

The objective of this study is to evaluate the educational competency of AEGD residents as an approach to recognize the missing link between the current dental education and the real-world demand for geriatric oral health professionals. The program trains six residents every year for three consecutive years. After one-year of training, we measured the effectiveness of clinical rotation and didactic experience including Age-Friendly practices. The hypothesis was that our AEGD program could advance every resident’s competence in geriatric oral care, and our program would lay a successful foundation to address the identified missing link for design of future PGD training.

## 2. Methods

### 2.1 Development of the AEGD program

Our featured AEGD residency program at the UTHealth School of Dentistry is one of the programs sponsored by Health Resources and Services Administration (HRSA) aiming to prepare the next generation of age-friendly practitioners (5). The Age-Friendly Health System (AFHS) framework is an initiative of the John A. Hartford Foundation and the Institute for Healthcare Improvement, in partnership with the American Hospital Association and the Catholic Health Association of the United States (17). It consists of evidence-based practices to improve wellness of older adults, known as the “4Ms": What **M**atters, **M**edication, **M**entation, **M**obility. In response to the growing aging population, HRSA provided funding to educational, community-based, and primary care institutions to improve the geriatrics workforce that are vital to deliver care in an age-friendly practice (18).

The AEGD program included didactic (three 4 h seminars) and four weeks of clinical education. Three seminars consisted of reviewing the process of natural aging, stereotypes and ageism, and the changes in the function of organs. During the seminars, faculty and residents also discussed in very detail how chronic health conditions may further impact the body as individuals live longer with chronic conditions. The third topic was about medications that may raise concerns in oral health such as opioids, anticholinergic, anti-psychotic drugs, as well as drugs used for diabetes and heart disease. Additionally, through clinical case reviews, the AEGD residents learned the most common chronic conditions in older adults and their impacts on oral health. Each resident was then assigned to a four-week clinical rotation in two Community Health Clinics (CHCs), one nursing home, and one memory care facility, respectively.

### 2.2 Assessment of the AEGD program

The program was evaluated each year for three consecutive years. The assessment tool was the same for the first two years but was modified in the third year to better evaluate residents’ level of knowledge and skills.

#### 2.2.1 Project year 1

The first group of six AEGD residents received a 5-question survey to establish a baseline for evaluation their knowledge in treating older adults with multiple health complexity. Due to the interruption of COVID-19, the residents were not able to have any hands-on clinical activities. However, they received 12 h geriatric education didactically and remotely every week. The educations were focused on science of aging, stereotypes and ageism, the impact of polypharmacy, and the physical and cognitive changes may have negative impact on oral condition. The clinical education was limited to clinical case presentations within the 4Ms framework, discussing treatment of choice and choice of dental materials, anesthetics and other person-centered clinical decisions based on each person’s needs and what Matters most to them.

#### 2.2.2 Project year 2

The second group of 6 residents participated in the same 5 question survey except this group had a short window of opportunity to treat older adults as it was planned in two CHCs, a community nursing home and a memory care. Each resident rotated in all three locations per week and the overall rotations took one month. After RCQI analysis of the second year, we noticed that the 5-question survey is not sufficient to evaluate clinical learnings with an emphasis on health conditions and the 4Ms. It was also noticed that weekly rotation to different sites does not offer sufficient continuity to see the outcome of treatments. Therefore, we assigned each resident to 4 consecutive weeks (one month) of participation in geriatric oral health education.

#### 2.2.3 Project year 3

The third group of six residents were enrolled to the program and participates in 10-question survey before and after the rotation. With the previous evaluation result, the project team added another five questions to the survey to become a 10-question survey. In year 3, all six residents had the entire duration of AEGD program to complete geriatric education and clinical rotation. Five additional questions allowed the project team to better evaluate the clinical advancement of the learners and the future application of the 4Ms in practice.

### 2.3 Data source and measurements

The AEGD program is a one-year post-graduate residency program to advance training in general dentistry. To be eligible for this study, residents must have graduated from an accredited dental school in the U.S or were accepted into the AEGD program as international residents. The program recruited six residents in each of 2019–2020, 2020–2021, and 2021–2022 and a total of 18 residents responded to pre- and post-rotation surveys. The study has been approved by the UTHealth Institutional Review Board.

The purpose of this study is to assess and evaluate the education and clinical skills of the AEGD residents, before and after the rotation. All residents participated in a 5-question survey before any exposure to the content of the program and the same 5-question after completion of rotations. The third cohort (2021–2022) of six residents has responded to five additional questions before and after the rotation. For questions 1, 7, 8, 9, and 10, the responses range from 1 = strongly disagree to 5 = strongly agree. For questions 2, 3, 4, 5, and 6, the responses range from 1 = strongly agree to 5 = strongly disagree. In all questions, a higher grade indicates that residents’ knowledge and confidence have been improved after the rotation. Residents’ personal identifiers were removed, and only aggregated outcomes were reported in this study.

### 2.4 Statistical analysis

**Figure 2:**
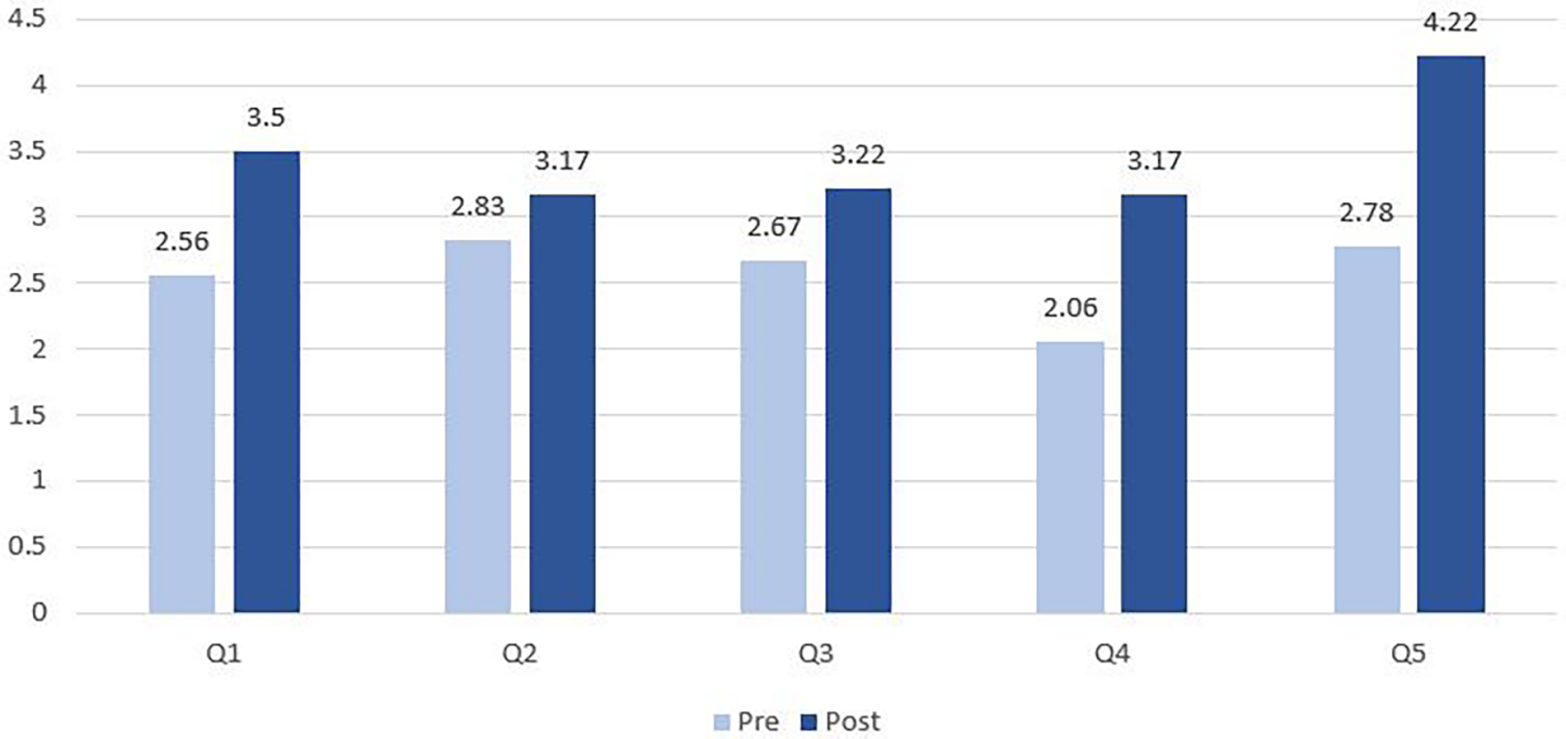
Pre- and post-rotation responses to the first five questions (18 residents).

**Figure 3:**
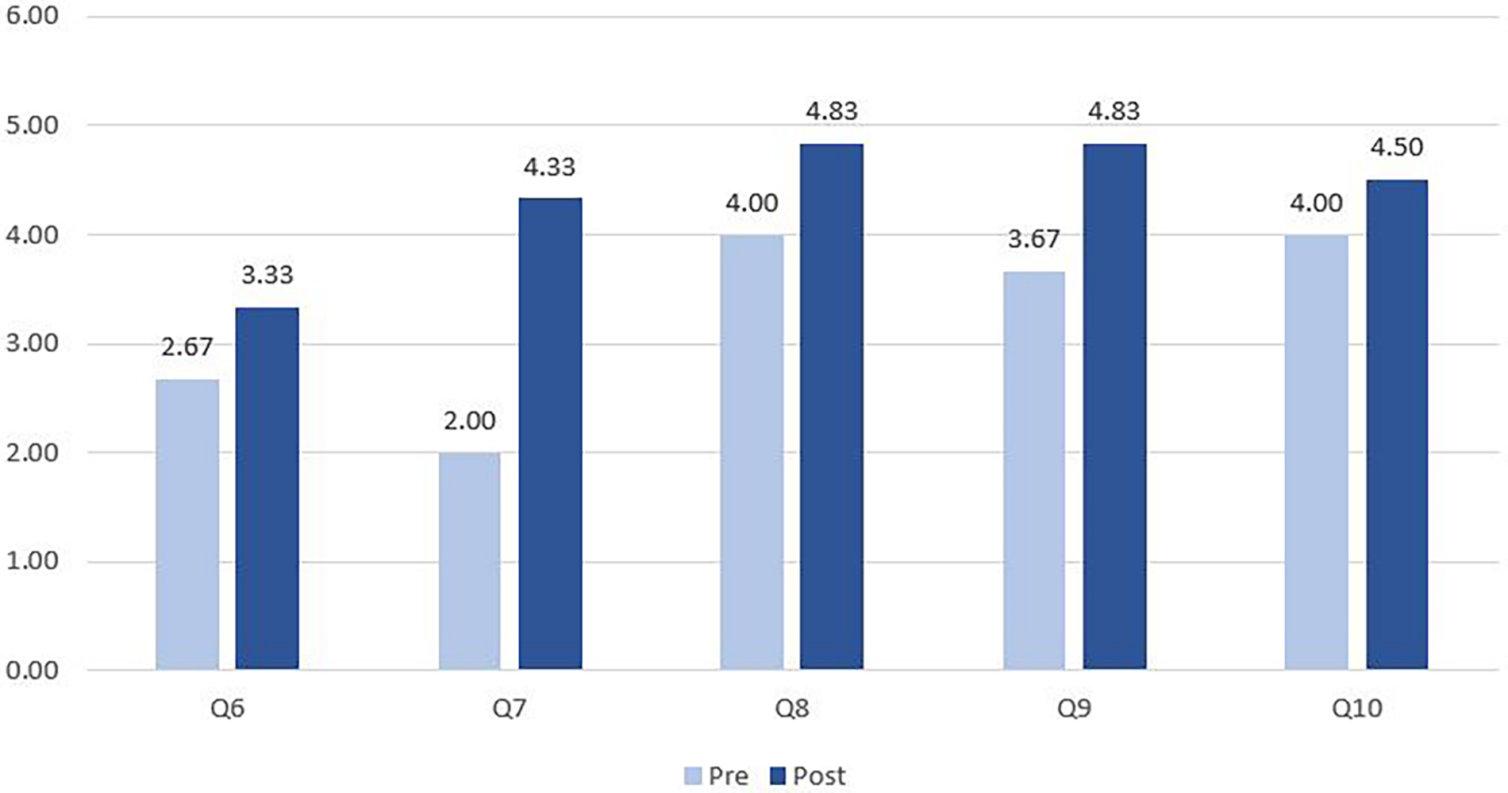
Pre- and post-rotation responses to the last five questions (6 residents).

A five-point Likert scale was used to rate each resident’s response to survey questions. We linked each resident’s responses to pre- and post-rotation surveys and then conducted Wilcoxon test with Fisher Exact modifications that better addressed our small sample (*N* = 18). Mean score, standard deviation, and *p*-value were presented in Table 1 to highlight changes in scores after residents completed the rotation. Mean scores were presented in Figure 2 and Figure 3 to show overall improvements in knowledge and confidence. Statistical analysis was performed by STATA v.16 and the significant level was set at *p* ≤ .05. The project has been approved by Institutional Review Board (#229293) of the UTHealth Houston.

**Table 1:** Differences in knowledge and confidence before (Pre) and after (post) rotation.

Survey question	*N*	Pre mean(std.)	Post mean(std.)	*p*-value
Q1. I am comfortable treating elderly with multiple health complexities.	18	2.56 (.25)	3.50 (.22)	0.011
Q2. Extraction of weak teeth is an acceptable treatment for the elderly to prevent future oral health problems and maintenance.	18	2.83 (.32)	317 (.09)	0.346
Q3. I do not feel I have adequate training to treat medically vulnerable elderly.	18	2.67 (.21)	3.22 (.24)	0.056
Q4. Elderly with physical and cognitive declined with behavioral management should see specialist in the filled.	18	2.06 (.26)	3.17 (.09)	0.001
Q5. Number of remaining teeth in elderly is unrelated to their rate of mortality.	18	2.78 (.17)	4.22 (.22)	<0.001
Q6. I am satisfied with the level of geriatric oral health education I have had, before my residency.	6	2.67 (.67)	3.33 (.33)	0.491
Q7. The 4Ms framework is applicable to all patients aged 65 and above.	6	2.00 (.00)	4.33 (.49)	0.015
Q8. I should always consider chronic conditions and related medication while planning dental treatments.	6	4.00 (.45)	4.83 (.17)	0.197
Q9. Patient’s cognition and the condition of oral health are always associated.	6	3.67 (.42)	4.83 (.16)	0.054
Q10. As a general dentist, I will integrate the 4Ms structure to all my geriatric patient’s treatment.	6	4.00 (.00)	4.50 (.22)	0.182

For questions 1, 7, 8, 9, and 10, the responses range from 1 = strongly disagree to 5 = strongly agree. For questions 2, 3, 4, 5, and 6, the responses range from 1 = strongly agree to 5 = strongly disagree. In all questions, a higher grade indicates that residents’ knowledge and confidence have been improved after the rotation.

## 3. Results

Table 1 illustrates 18 residents’ responses to five original questions and 6 residents’ responses to five newly added questions. Before the rotation, most students did not feel comfortable treating older adults with multiple health complexities (mean = 2.56). Likewise, they did not agree that older adults with physical and cognitive decline should see specialist (mean = 2.06) and the 4Ms framework is applicable to all patients aged 65 and above (mean = 2.00). After the rotation, all residents became more likely to have confidence and knowledge toward treating geriatric populations (*p* < 0.05 for Q1, Q4, Q5, and Q7). Although there is no statistical significance in changes in other questions, the mean scores were increased in all questions.

Figure 2 demonstrates the changes in competence of 18 residents before and after rotations by bar charts. Among the first five questions, Q2 has the lowest post-rotation score and the smallest variation from 2.83 to 3.17 (*p *= 0.346). Q5 has the highest post-rotation score and the largest variation from 2.78 to 4.22 (*p *< 0.05).

Figure 3 demonstrates the changes in competence of 6 residents (the third cohort) before and after the rotation. Among the last five questions, Q6 has the lowest score after the rotation and the smallest variation from 2.67 to 3.33 (*p *= 0.491). Q7 has the largest variation from 2.00 to 4.33 (*p *< 0.05). Considering these findings, more training about extraction of weak teeth and geriatric oral health is highly recommended.

## 4. Discussion

### 4.1 Overall findings

The AEGD program as a post-graduate training was intentionally designed to enable residents to provide treatments based on the 4Ms model to meet oral health needs of older adults (19). The study sought to evaluate the current knowledge of age-friendly practices of recently graduated general dentists such as how comfortable they felt about treating medically vulnerable older adults with or without dementia. After the rotation, the study further tested residents’ confidence and competence in practicing under the 4Ms framework. Each cohort of residents in the initial survey were not sure if they had sufficient training in the past or felt unsure about how useful the training was if they had it. After participating in our AEGD program with didactic education and clinical rotations, more residents felt comfortable treating older adults and integrating the 4Ms to their treatment (based on their responses to Q1, Q7 and Q10). They also learned to consider chronic conditions and cognition of older adults while treating dental issues (based on Q2, Q4, Q5, Q8, and Q9). Third, more residents admitted that they did not have adequate training to treat medically vulnerable older adults (based on Q3 and Q6). They realized the threads within curriculum and isolated lectures are not sufficient for them to treat patients with special needs as defined by the Commission on Dental Accreditation (CODA) standard 2–25: Patients whose medical, physical, psychological, or social situations make it necessary to consider a wide range of assessment and care options (7). The finding of this study has clearly demonstrated that geriatric oral health education is missing from many dental education programs.

### 4.2. Meet the needs of aging population

These positive improvements in residents’ competence are critical because as discussed earlier, over 22 million older adults will need special care by 2030 due to medical vulnerability, chronically being in poor health, and decline in quality of life (20). For example, 6.5 million Americans aged 65 and older are living with Alzheimer’s disease and this number will escalate to 12.7 million by 2050 (21). In addition, every 40 s, someone has stroke in the US and to date, there are approximately 795,000 stroke individuals of which about 610,000 cases are first time or new strokes (22). Almost all medications to treat Alzheimer’s disease, cardiovascular diseases, and stroke will cause dry mouth which is the single most important factor for dental caries due to creating a low acidic PH (23). Poor oral health is also seen in older adults due to factors including physical changes by getting older, poor nutrition, hormonal changes, and polypharmacy such as anticholinergic drugs (24). Physical changes like xerostomia are caused by decreased salivary flow and difficulty in maintaining a proper healthcare regimen like brushing and flossing and all these could be exaggerated by polypharmacy (25).

Medically vulnerable elders need special protection and different treatments from general population (26). Consequently, expanding geriatric oral health training in dental education programs is timely and necessary to meet the increasing demand of geriatric populations. Although more than 90% of 56 U.S. dental schools indicated that they have compulsory classes about medical problems of older adults, other topics such as nutrition, drug-induced dental issues are less likely to be covered in the training (9, 27). Geriatric dentistry like pediatric dentistry or other disciplines in oral health requires a designated training track within dental education. Without additional didactic and clinical geriatric education, general dentists graduating from dental schools will not be capable to provide care to geriatric population. Additionally, older adults with dry mouth may be seen in the form of bad breath, increased caries, poor retention of oral prostheses, difficulty eating, discomfort, fungal infections, and ulcerations. These poise a challenge to individual’s health outcome and interpersonal relationship. Therefore, a comprehensive dental training that covers geriatric care is needed. In short, this study suggested that all general dentists be trained to conduct person-centered treatment plans adopting the 4Ms framework. Advanced Education in General Dentistry should respond to the changing needs of older adults with complex chronic conditions. Integrating aging-friendly concept into undergraduate dental programs might also be helpful to bring more awareness of this specific skill and scope of practice for future dental professionals.

### 4.3 Limitations

There is no standardized training to guide faculty members to establish a residency program integrating geriatric care and evaluate the effectiveness of such a program (10). The project team adopted the Rapid Cycle Quality Improvement model to adjust the curricula to incorporate more information and practice experiences along the way. As a result, the sample size in the Year 3 is insufficient to be generalized to other populations. However, combination of evidence-based practice (e.g., 4Ms model) and trainee-generated data has enabled us to design more appropriate measurements even during the pandemic. More studies in program evaluation will provide a better insight into how to teach students and residents to determine the best treatment for frail and functionally dependent older adults.

## 5. Conclusion

Recent general dentist graduates who enrolled in our AEGD program from various dental institutions (within or outside of the U.S) felt they are not prepared to treat older adults with multiple chronic conditions. Our study demonstrated the improvements in their confidence and knowledge to provide age-friendly care. Strengthening dental education in both didactic and clinical skills to assess and treat older adults with complex needs is highly recommended in response to the increasing number of older adults. Strengthening the CODA standard and faculty capacities to establish more geriatric oral health programs can also help close the gap.

## Data Availability

The raw data supporting the conclusions of this article will be made available by the authors, without undue reservation.
